# The role of motivational components in metamotivational monitoring in medical students: a mixed method study

**DOI:** 10.1186/s12909-023-04081-y

**Published:** 2023-02-13

**Authors:** Ali Norouzi, Dean Parmelee, Mohammad Shariati, Saiideh Norouzi, Maryam Alizadeh

**Affiliations:** 1 Education Development Center (EDC) and School of Medicine, Zanjan University of Medical Sciences, Zanjan, Iran; 2 Medical Education, Psychiatry & Pediatrics, Wright State University Boonshoft School of Medicine, Dayton, OH USA; 3 Department of Medical Education and Department of Community Medicine, School of Medicine, Tehran University of Medical Sciences, Tehran, Iran; 4 Department of Nursing, Abhar School of Nursing, Zanjan University of Medical Science, Zanjan, Iran; 5 Department of Medical Education and Education Development Center (EDC), Tehran University of Medical Sciences, Tehran, Iran

**Keywords:** Motivation, Self-regulation, Medical student, Medical education

## Abstract

**Background:**

In metamotivational monitoring, students try to identify the declined motivational component in order to regulate their motivation. There is scarcity of evidence on which motivational components are targeted by the medical students when they use each motivational regulation strategies. This study aims were identifying motivational components in motivational regulation process, developing a measurement tool and, testing the predictive relationship between the motivational components and motivational regulation strategies.

**Methods:**

This exploratory sequential design mixed method study is part of a project has been started from 2018 with medical students at Tehran University of Medical Science. First, in a qualitative study conducting a semi-structured in-depth interview, the motivational components were explored. The interviews continued until saturation of data. Then, in a psychometric study the validity and reliability evidence of questionnaire obtained. In the quantitative study, applying the convenience sampling method, 508 students completed the questionnaires. Predictive relation between the motivational regulation strategies and motivational components was assessed utilising Structural Equation Modelling. Path coefficients, T-Value, and R^2^ index were reported by SmartPLS software.

**Results:**

In the Exploratory Factor Analysis of Motivational Components Questionnaire (MCQ), 6 factors were discovered that explained 74% of the total variance. All paths in seven models of SEM showed a T-Value above 1.96 that means there is a significant correlation between all strategies and components. In examining the predictive relationships, each of the four components of self-efficacy, intrinsic value, self-relevant value and promotion value were specifically predicted by two motivational regulation strategies.

**Conclusions:**

Evidence of validity and reliability of the MCQ indicates that this questionnaire can be used in medical education contexts. Health Profession Educators can improve the academic motivation of students by identifying one or more declined motivational component and teaching specific motivational regulation strategies. It is recommended to hold training courses on motivational regulation strategies for medical school faculty, study-skills advisors, and students.

**Supplementary Information:**

The online version contains supplementary material available at 10.1186/s12909-023-04081-y.

## Background

Metamotivation is how people monitor and control their motivational states to achieve their goals [1]. Research findings indicate that some motivated students utilize specific strategies to enhance their motivational states [2], and consequently, improving their performance [3, 4]. A common issue is that learner role often gets skipped in controlling and regulating motivation in the context of medical education [5]. We know from the literature, outside the field of HPE, students are able to regulate their motivational components utilizing motivation regulation strategies [6]. The contextual aspects of medical education, such as basic science and clinical phase as well as highly specific educational environment, do not exist in other scientific disciplines. From this point of view, Norouzi et al. found that medical students use seven motivational regulation strategies, including regulation of value, regulation of situational interest, self-consequating, environmental structuring, promotional situational awareness, preventional situational awareness, and regulation of relatedness to monitor and control their academic motivation [7]. The identification of motivational regulation strategies is a significant step in understanding the metamotivational process, but another step, is identifying motivational components which are targeted by these motivational strategies [8].

A well-known and comprehensive model of metamotivation, developed by Miele & Scholer, define metamotivation as two reciprocal processes: A. Metamotivational monitoring; evaluating whether the person has selected the proper level (quantity) and type (quality) of motivation to perform his tasks. B. Metamotivational control; using the results of the monitoring phase and applying suitable strategies for adapting or changing the motivation [6, 9]. In the metamotivational monitoring phase, the motivational components play an essential role. When students feel like leaving a task, they monitor the motivational components to recognize which have declined and caused such a feeling. Also, when they think they have a wrong mindset or orientation for a task, they monitor their motivational components and alter them commensurate with the task at hand [8].

Considering three motivational theories (expectancy-value theory, self-determination theory, and regulatory focus theory), these researchers specified six motivational components include self-efficacy, intrinsic value, self-relevant value, external value, promotion value, and prevention value involved in the metamotivational model. They emphasized that to establish the nature of these motivational components, a survey should be designed in which not only various types of motivational regulation strategies are explored, but also the *reasons* for using strategies are determined and lead to clarity on the motivational components using factor analysis of responses [8]. On the other hand, it is expected that based on this model, every motivational component is predicted directly using a limited number of motivational regulation strategies.

Therefore, it seems that for the following reasons there was a need to identify the motivational components and design its measurement tool:

1. Raising self-awareness of the status of motivational components as a trigger to use strategies,
2. Identifying the influenced motivational components to choose the right strategy and
3. Expanding the studies in the field of metamotivation.

Norouzi et al. explained the motivational regulation strategies and designed Metamotivational Strategies in Medical Students Questionnaire (MSMQ) to use in the medical education context [10]. However, the question remains, which underlying components of motivation is targeted by the medical students when taking advantage of these strategies? To answer this question first, we identified the motivational components in motivational regulation process in medical students and developed a measurement tool. Then, we tested the predictive relationship between the motivational components and motivational regulation strategies.

## Methods

### The aim, design and setting of the study

This exploratory sequential design mixed method study [11] is part of a big project has been started from 2018 with medical students at Tehran University of Medical Science. In this manuscript, we present the results of two phases (Table 1). A. Identifying motivational components in motivational regulation process and developing its measurement tool, B. Investigating the predictive relationship between the motivational components and motivational regulation strategies.

**Table 1:** The steps of study

Phases	Steps		Type of study	Sampling method
Phase A	1	Collecting the reasons of using motivational regulation strategies	Qualitative	Purposeful
	2	Designing the Motivational Components Questionnaire (MCQ) items	Psychometric	Purposeful and convenience
	3	Investigating the evidence of content validity of MCQ		
	4	Examining the evidence of response process validity of MCQ		
	5	Examining the evidence of structural validity and reliability of MCQ		
Phase B	6	Assessment of predictive relation between the motivational regulation strategies and motivational components	Quantitative	Convenience

### Study steps

#### Phase A

##### Step 1. Collecting the reasons for using motivational strategies

First, in a qualitative study, the motivational regulation strategies used by the medical students were explored. Participants were selected via purposive sampling. This study was conducted through a semi-structured, in-depth interview in which participants were asked to think about their real educational experiences where they felt their motivation states were challenged in the educational environment. Next, they explained the strategies used to monitor, sustain or control their learning motivation. After a reference to a strategy by the student, the researcher further probed for the *reason* behind the strategy (i.e., why do you use this strategy?). At the end of each interview, the *reasons* for using motivational regulation strategies were gathered. The interviews continued until saturation of data.

To ensure credibility, the principal investigator was continuously involved in the research topic for 8 consecutive months and used the methods of bracketing and member check. One expert out of the research team reviewed the analyses to ensure confirmability. To ensure data dependability, 10% of the data were encoded by two independent individuals. Furthermore, to achieve transferability, maximum variation was considered in sampling in terms of gender and phase of education. Further details of this part of study has been published in another article in Journal of Education and Health Promotion [12].

##### Step 2. Designing the Motivational Components Questionnaire (MCQ) items

A panel of five experts in medical education and psychology was formed. In this session, the *reasons* collected from the interviews were categorized, and questionnaire items were compiled according to the common concept of each category of reasons.

##### Step 3. Investigating of the evidence of Content Validity in MCQ

Fifteen experts in the field of medical education and psychology were enlisted to score the Content Validity Index (CVI) and Content Validity Ratio (CVR) indices. They were asked to rate each item's relevance, clarity, and simplicity on a four-point Likert scale to allow calculation of the CVI index [very relevant (4), relevant (3), somehow relevant (2), irrelevant (1)]. The CVI was then calculated using the Waltz and Bausell formula, for which the index is acceptable when the average score obtained from all items is higher than 0.79 [13].

$$\mathrm{CVI}=\frac{(\mathrm{Number}\;\mathrm{of}\;\mathrm{experts}\;\mathrm{who}\;\mathrm{gave}\;\mathrm{score}\;3\;\mathrm{or}\;4\;\mathrm{to}\;\mathrm{each}\;\mathrm{item})}{(\mathrm{Total}\;\mathrm{number}\;\mathrm{of}\;\mathrm{experts})}$$

The CVR was calculated using the Lawshe formula [14]. Experts were asked to categorize each question according to a 3-point Likert scale of "item is necessary," “item is useful but not necessary,” and “item is not necessary.” Then, CVR was calculated using the following formula. Taking into account the number of experts (*N* = 15) and the values in the Lawshe table, the items with CVR < 0.49 were eliminated from the questionnaire (N: total number of experts; Ne: number of experts who selected the item “necessary”)

$$CVR=\frac{Ne-\frac{N}{2}}{\frac{N}{2}}$$

##### Step 4. Examining the evidence of response process validity in MCQ

In this step, six medical students were interviewed. The aim of this step was to compare the students’ interpretation of each item with the intended purpose of the designers and assuring congruence through modification as needed. Students' perceptions about each item were investigated with “thinking aloud” and “concurrent verbal probing” methods.

##### Step 5. Examining the evidence of structural validity and reliability in MCQ

The Exploratory Factor Analysis (EFA) was used to evaluate the instrument and gather the evidence of structural validity. The convenience sampling method was also applied. The instrument was designed using the ePoll, an online test maker application, as an online questionnaire, and its link was sent to the medical students through social networks. Considering the number of items and the assumptions of the EFA, the minimum sample size was determined to be 120 (5 subjects per item). 224 students completed the questionnaire. The data were analyzed by the IBM SPSS Statistics 26 for Windows (IBM Corp. Armonk, NY). The Kaiser–Meyer–Olkin (KMO) index (> 0.7) was used to evaluate the adequacy of the sample size, and Bartlett's test of sphericity was used to determine the factorability of the data. Also, the Principal Component method was used with Varimax rotation to extract the components, and the items with a factor load greater than 0.4 were preserved. To identify the tool’s reliability evidence, Cronbach's alpha coefficient and Intraclass Correlation Coefficient (ICC) analysis were used. After EFA and determination of the tool’s structure, the Cronbach's alpha of subscales and the overall instrument were calculated. The final questionnaire was given twice to 23 students with a two-week interval to calculate the ICC. Common cut-off points for ICC assessment were used; > 0.90 (excellent), 0.75–0.90 (good), 0.60–0.75 (moderate), and < 0.60 (poor) [15].

#### Phase B

##### Assessment of predictive relation between the motivational regulation strategies and motivational components

In this step MCQ and Metamotivational Strategies in Medical Students Questionnaires (MSMQ) [10] were used. The important question was to identify which motivational component is targeted *when* using any of the seven strategies?

The students were asked to answer the questions related to each motivational strategy i.e., determine how much they use that strategy and then, determine their reasons (by answering MCQ questions) *when* using that specific strategy. Therefore, seven online questionnaires were designed separately and sent to seven different groups of medical students (One questionnaire for each strategy). In this stage, we formed seven structural models (see Additional file 3: Appendix 3). Based on the 10-times rule [16] in the Partial Least Squares (PLS) method, the minimum sample size was estimated 60 per model.

The SmartPLS software (SmartPLS 2.0.M3. Hamburg) analyzed collected data. The validity was assessed using convergent validity, divergent validity, composite reliability, and Cronbach's alpha coefficient. In the PLS, the convergent validity includes the Average Variance Extracted (AVE) of subscale questions. Accordingly, convergent validity is confirmed when the AVE is more than 0.5. Divergent validity was tested via the cross-loading method. According to this index, if the item related to a subscale has a higher factor load on another subscale, it will be removed from the model. Composite reliability and Cronbach's alpha coefficient were also confirmed with a reliability greater than 0.7.

The predictive relationship between the variables was calculated through SEM. Path coefficient, T-Value, and *R*^2^ index were used to investigate the predictive relations between latent internal and external variables. When the T-Value of a path is greater than 1.96, it is confirmed at a significance level of < 0.05. The statistic *R*^2^ indicates the level of changes in the endogenous variable that the exogenous variable can predict. Commonly, for the dependent variable in the structural model, the values ​​of 0.19, 0.33, and 0.67 have been described as weak, medium, and significant, respectively. However, if the latent endogenous variable is affected by a small number of the exogenous variables (one or two), the medium values ​​of *R*^2^ could also be accepted [17, 18]. Considering that, in the present study, all latent endogenous variables were affected by only one exogenous variable, the values of *R*^2^ higher than 0.33 were deemed acceptable.

## Results

### Phase A: identifying motivational components and designing its measurement tool

After interviewing the medical students, the data were analyzed, and 207 phrases were gathered as the specific reasons for using the motivational regulation strategies. In the expert panel, the conceptually related reasons were categorized into 24 categories. Then, an item was formulated for each group of reasons, which conceptually covered all the reasons in that category. The responses to the items were also scored on a 5-point Likert scale (never, rarely, sometimes, usually, and always). In the analysis of the CVI index, the instrument gained a score above 0.79 for transparency, relevancy, and simplicity. The CVR index also gained a score above 0.49 for all items, and none of the items was eliminated from the study (see Additional file 1: Appendix 1).

Students’ interpretations confirmed the evidence of response process validity. In the EFA, the KMO test obtained a score of 0.89, and Bartlett's test of sphericity was significant. This finding indicates that the sample size was adequate and the data are factorable. The total variance explained table shows that six factors with an eigenvalue greater than one could estimate 74% of the variance. The result indicated that, the items of the first factor refer to reasons such as making the academic achievement enjoyable and attractive. This factor was entitled “intrinsic value.” Also, conceptually, the items of factor 2 referred to the student's efficacy in confronting academic challenges. This factor was entitled “self-efficacy.” In the items of factor 3, the students tried to provide reasons for using the motivational regulation strategies, where they referred to the prevention of problematic situations. So, this factor was entitled “prevention value.” On the other hand, in the items of factor 4, the students tried to provide reasons for using the motivational regulation strategies, where they referred to the academic achievements. This factor was, thus, called the “promotion value.” In addition, the reasons referred to in the items of factor 5 were conceptually related to achieving academic values and benefits. This factor was entitled “self-relevant value.” Finally, the items of factor 6, which included concepts such as awards and rewards, were entitled “external value.” The lowest factor load was 0.61, and the highest was 0.89. The alpha coefficient of the overall instrument was 0.92. The alpha coefficient of the subscales is provided in Table 2. Calculation of the ICC index indicated that all subscales are at the “excellent” (higher than 0.90) and “good” (0.75–0.90) levels.

**Table 2:** Factor loading of items, Cronbach’s alpha coefficient and ICC values of MCQ subscales

Items	Factors						Cronbach’s alpha	ICC
	1	2	3	4	5	6		
So that studying my lessons becomes pleasant	0.88						0.92	0.83
So that my presence in the educational environment becomes pleasant	0.82							
So that I continue my studies with the highest interest and joy	0.81							
So that I confront academic challenges with interest and joy	0.78							
So that I confide of my efficacy in my academic major		0.86					0.90	0.92
So that I enhance my academic capabilities		0.85						
So that I enhance my capabilities to confront academic challenges		0.84						
So that I improve my belief in my potentials		0.77						
So that I won't be a weak student in my academic major			0.89				0.87	0.83
So that I will not face academic failure			0.85					
So that my academic curve will not be descending			0.81					
So that I will not be a low literacy student			0.64					
So that I continue my academic achievements				0.84			0.83	0.87
So that I achieve increasing success in my studies				0.75				
So that I progress in my academic major				0.73				
So that my academic achievements will not be interrupted				0.68				
So that I fulfill the valuable potentials of my major					0.79		0.84	0.90
So that I benefit from the values of my discipline					0.71			
So that I make the most out of my presence in the academic environments					0.70			
So that I benefit from the educational courses					0.63			
So that I would rank high among the students of the class						0.79	0.85	0.81
So that I show a good academic reputation in college						0.71		
So that I get good marks in my courses						0.70		
So that I will be acknowledged and admired						0.61		

### Phase B: predictive relation between the motivational regulation strategies and motivational components

Using convenience sampling method, 508 medical students completed the questionnaires. The descriptive statistics of those who completed the seven questionnaires can be observed in Table 3.

**Table 3:** Frequency and percentage of participants’ gender and phase of education in structural models

Models	Gender		Phase of education				Total
	Female	Male	Basic sciences	Physiopathology and semiology	Clerkship	Internship	
Regulation of value → motivational components	38 (47.5%)	42 (52.5%)	61 (76.3%)	9 (11.3%)	8 (10%)	2 (2.5%)	80
Regulation of situational interest → motivational components	29 (43.3%)	38 (56.7%)	54 (80.6%)	5 (7.5%)	4 (6%)	4 (6%)	67
Regulation of relatedness → motivational components	36 (55.4%)	29 (44.6%)	37 (56.9%)	13 (20%)	11 (16.9%)	4 (6.2%)	65
Promotional situational awareness → motivational components	46 (68.7%)	21 (31.3%)	40 (59.7%)	11 (16.4%)	11 (16.4%)	5 (7.5%)	67
Preventional situational awareness → motivational components	47 (46.5%)	54 (53.5%)	73 (72.3%)	6 (5.9%)	17 (16.8%)	5 (5%)	101
Environmental structuring → motivational components	41 (61.2%)	26 (38.8%)	30 (44.8%)	18 (26.9%)	10 (14.9%)	9 (13.4%)	67
Self-consequating → motivational components	46 (75.4%)	15 (24.6%)	41 (67.2%)	12 (19.7%)	2 (3.3%)	6 (9.8%)	61
Total	283 (55.7%)	225 (44.3%)	336 (66.1%)	74 (14.5%)	63 (12.4%)	35 (6.8%)	508

The results of model validity assessments are provided in Additional file 2: Appendix 2. Divergent validity assessment showed that only in the model of “environmental structuring” “motivational components”, item 4 of the subscale of “prevention value” had a factor load of 0.45, and 0.55 on Self-efficacy subscale. Therefore, this item was eliminated from the model, and the model was formulated again. In other models, all factor loads were confirmed.

The AVE index of all subscales in the seven models was above 0.5. Composite reliability and Cronbach's alpha coefficient of all subscales were also above 0.7. These results indicated that both instruments have best evidences of reliability and validity.

The path coefficients and the standardized loading factors for all structural models are presented in Additional file 3: Appendix 3. Investigation of the significance of path coefficients and loading factors indicated that the T-Value of all paths in all seven models was above 1.96. This showed a positive and significant correlation between all motivational regulation strategies and all motivational components. Table 4 shows the path coefficients, T-Value and *R*^2^ values in all models.

**Table 4:** Path coefficients, significance of path coefficients, R2 values in the predictive relationship between and motivational components

Structural Models	Structural paths	Path coefficients	T-value	R^2^
Model 1	Regulation of value → Self-efficacy	0.64	9.77	0.41
	Regulation of value → External value	0.27	2.84	0.08
	Regulation of value → Intrinsic value	0.72	12.14	0.52
	Regulation of value → Self-relevant value	0.73	10.27	0.53
	Regulation of value → Promotion value	0.53	5.77	0.28
	Regulation of value → Prevention value	0.32	3.56	0.10
Model 2	Regulation of situational interest → Self-efficacy	0.51	7/33	0.26
	Regulation of situational interest → External value	0.40	4/70	0.16
	Regulation of situational interest → Intrinsic value	0.72	18/43	0.52
	Regulation of situational interest → Self-relevant value	0.50	6/91	0.25
	Regulation of situational interest → Promotion value	0.58	7/76	0.56
	Regulation of situational interest → Prevention value	0.48	8/13	0.23
Model 3	Regulation of relatedness → Self-efficacy	0.37	4.57	0.14
	Regulation of relatedness → External value	0.29	3.75	0.08
	Regulation of relatedness → Intrinsic value	0.43	5.66	0.19
	Regulation of relatedness → Self-relevant value	0.52	6.84	0.27
	Regulation of relatedness → Promotion value	0.54	7.10	0.29
	Regulation of relatedness → Prevention value	0.34	3.59	0.11
Model 4	Promotional situational awareness → Self-efficacy	0.58	6.56	0.34
	Promotional situational awareness → External value	0.44	4.97	0.19
	Promotional situational awareness → Intrinsic value	0.40	4.01	0.16
	Promotional situational awareness → Self-relevant value	0.51	4.93	0.26
	Promotional situational awareness → Promotion value	0.66	8.95	0.44
	Promotional situational awareness → Prevention value	0.42	5.78	0.17
Model 5	Preventional situational awareness → Self-efficacy	0.34	3.42	0.12
	Preventional situational awareness → External value	0.48	6.52	0.23
	Preventional situational awareness → Intrinsic value	0.17	2.21	0.03
	Preventional situational awareness → Self-relevant value	0.24	2.28	0.06
	Preventional situational awareness → Promotion value	0.31	3.06	0.10
	Preventional situational awareness → Prevention value	0.41	4.76	0.16
Model 6	Environmental structuring → Self-efficacy	0.35	6	0.12
	Environmental structuring → External value	0.28	2.75	0.08
	Environmental structuring → Intrinsic value	0.50	6.07	0.25
	Environmental structuring → Self-relevant value	0.57	9.72	0.33
	Environmental structuring → Promotion value	0.39	4.27	0.15
	Environmental structuring → Prevention value	0.40	5.2	0.16
Model 7	Self-consequating → Self-efficacy	0.38	5.40	0.14
	Self-consequating → External value	0.40	5.38	0.16
	Self-consequating → Intrinsic value	0.45	6.09	0.22
	Self-consequating → Self-relevant value	0.46	5.59	0.21
	Self-consequating → Promotion value	0.42	5.73	0.18
	Self-consequating → Prevention value	0.37	5.48	0.14

The regulation of value predicted 41%, 52%, and 53% of the variations of self-efficacy, intrinsic value, and self-relevant value, respectively. The regulation of situational interest also predicted 51% and 56% of the intrinsic value and promotion value, respectively. Promotional situational awareness predicted 33% and 43% of self-efficacy and promotion value. The environmental structuring was a predictor of the self-relevant value with a value of 33%.

The two components of external value and prevention value could not be predicted well by the motivational regulation strategies. The three strategies of regulation of relatedness, preventional situational awareness, and self-consequating could not predict the motivational components at acceptable level.

## Discussion

The present study identifies the motivational components targeted by motivation regulation strategies, creates a tool to measure them, and determines a predictive relation between the motivational components and motivational regulation strategies. The high percentage of the total variance explained in the EFA as well as the lack of cross factor loading between the items indicates that the factors have been appropriately explained and are major and distinguished components in this process. Also, there is proper evidence of the reliability and validity of the results obtained using this instrument. Motivational components identified in this study are entirely adapted to the taxonomy of motivational components in the Miele and Scholer's metamotivational model [8].

The results of analyzing the validity of models in Phase B indicated that both instruments provide best evidence of structural validity and reliability. Investigation of convergent validity in all models showed that the motivational components and motivational regulation strategies had been appropriately explained. On the other hand, the divergent validity of the items indicated that all items had a proper factor load in their subscale. In addition, a positive and significant relationship between all motivational regulation strategies and motivational components in the seven models was another essential result that indicated the reason for the comprehensive explanation of strategies and components.

The findings of this study regarding the intrinsic value mainly were in line with the expectations of models of Miele and Scholer. These researchers speculated that this motivational component is specifically affected by the regulation of value strategy [8]. We should notice that the metamotivational approach is built on the assumption that students have awareness to why they engage in certain strategies. it is relevant to look at people’s beliefs, and it is likely true that students often do have good insight into their motivation. However, like metacognitive process they can occur implicitly or automatically [9].

This study also showed that the regulation of value and regulation of situational interest both predict the intrinsic value at acceptable level. The intrinsic value originates from the self-determination theory [19]. The highest quality of motivation is intrinsic and autonomous types of motivation which is related to deep learning and better performance of learners [19, 20].

Self-efficacy is the student’s belief in his/her potentials for the successful performance of tasks [21]. It is one of the most potent predictors of academic effort, and perseverance [22]. In his study, Wolters concluded that the students could affect the self-efficacy through their motivational regulation strategies [2]. Studies in medical education also indicates that self-beliefs and self-efficacy facilitate the learning and development of medical students [22]. Pelaccia mentioned the perceived self-efficacy as one of the main components of effort, regulation, perseverance, and management of academic performance in medical students [23]. According to the Miele and Scholer's model, it is expected that efficacy self-talk and proximal goal setting could predict the self-efficacy [8]. This study demonstrates that these two strategies of regulation of value and promotional situational awareness could correctly predict the self-efficacy. This is even though the results of Norouzi et al. and Wang and Wolters indicate that the efficacy management strategy could not serve as a comprehensive strategy for metamotivational monitoring [2, 10, 24].

The theoretical basis of the two components of promotion value and prevention value is regulatory focus theory. Predominantly promotion-focused individuals state their goals as ideals and use eager strategies to achieve their goals. On the other hand, predominantly prevention-focused individuals take their academic goals as tasks and responsibilities and prefer vigilant strategies to achieve their academic goals [25]. It seems the medical students who think of their promotion and development, or those who are sensitive to inability in their academic improvement, target their promotion value in metamotivational monitoring. On the other hand, the students think about escaping from illiteracy, not getting the lowest marks, and not failing exams, seek to affect their prevention value. The predictions of the promotion value were relatively in line with the Miele and Scholer's model [8]. The present study results indicate that the two strategies of regulation of situational interest and promotional situational awareness can strongly predict this component. Even though the Miele and Scholer's model use mastery self-talk strategy as the likely predictor of prevention value [8], the results of this study indicated that no one of the motivational regulation strategies could predict this component at acceptable level (In Norouzi et al., promotional/ preventional situational awareness, as more comprehensive strategies, replaced regulation of mastery/performance goals [10]).

The self-relevant value is equivalent to identified and integrated regulation in the self-determination theory [8]. Using some strategies, the medical students try to remind themselves of the importance and suitability of medicine and educational factors to obtain a professional identity and become aware of the values of their field of study. In Miele and Scholer's model, the regulation of value has been taken as the predictor of self-relevant value. The results of this study have affirmed this idea. The two strategies of regulation of value and environmental structuring could predict 53% and 32% variations of the self-relevant value, respectively. The external value is also equivalent to external and introjected regulation in self-determination theory [8]. The individual indicates the reasons for doing things such as receiving rewards, escaping punishment, avoiding shame, etc. [19]. In the present study, the external value was not adequately predicted by any motivational regulation strategies. However, according to Miele and Scholer's model, it was expected that there would be a predictive relation between this component and self-consequating.

Despite their significant relationship with all motivational components, the three strategies of regulation of relatedness, preventional situational awareness, and self-consequating could not predict a substantial share of one of the motivational components alone. The finding enhances the likelihood that these three strategies could be used simultaneously with other strategies to improve effectiveness. That’s because, according to Miele and Scholer, there are strategies that increase the likelihood of inclusive engagement with tasks [9]. Using the regulation of relatedness and other motivational regulation strategies, medical students are likely to meet two or more academic goals at a time, thereby managing their motivation. For example, when a medical student tries to enter clinical education in hospital departments with a group of friends, he tries to both increase his relations with friends as well as his interest in attending that department by creating a fun atmosphere in the educational environment, so that he could strengthen his intrinsic motivation.

### Implication of this study

In this study, a tool with acceptable validity and reliability evidence was designed that researchers can use in their future studies in the field of metamotivation. This tool can be used by educationists in order to know the motivational components of students and based on the obtained information, targeted educational interventions can be designed. Also, this tool can be used for students' self-awareness of motivational status. Using the information obtained from the second phase of the study, psychologists and educational counselors can suggest motivational strategies which fit to students motivation states.

### Limitations and strengths

Real experiences of medical students were obtained through deep interviews, it can therefore be claimed that none of the identified components include an abstract aspect, and none have been inferred from the personal opinions and perceptions of the students. On the other hand, we tried to evaluate and prove the nature of the interview results through EFA.

The focus of this study was on medical students. Therefore, there is doubt in generalizing the findings of this study to other medical and non-medical students. Considering that we were looking for the ultimate reason for using strategies in phase 1; It was not easy for students to express these reasons that may have affected the identified components. It should also be noted that the interviews were held virtually due to the covid 19 pandemic. It is possible that conducting face-to-face interviews will make more and better-quality data available to the researcher.

Other limitation of our study is its relatively small sample size for the estimation of each structural model in Phase B. Although the minimum sample size was calculated based on the assumptions of SEM, it is evident that the higher the number of samples, the better the generalizability of the results.

## Conclusion and recommendations

The identification of motivational components is a very fundamental idea in the Miele and Scholer's model. This idea is a very significant effort for making metamotivational studies more purposeful. The results of this study indicate that the six motivational components of the Miele and Scholer's model are the key and essential components in this process. Although the expectations of the metamotivational model as to the predictive relation between the strategies and components were not fully met, in this study, the fundamental assumption of the metamotivational model, i.e., the direct relationship between one or two motivational regulation strategies and the motivational components was proven.

Future studies are recommended for the identification of the phenomenological experiences of the six mentioned components. The discovery of the structural relationship between these components and the medical students' desire and intention of the medical students to perform their academic tasks is a need. It is expected that future studies investigate the effect of simultaneous use of several motivational regulation strategies on the motivational components. If the goal is to use this scale in other research, it seems that it might be useful to have an additional study that links strategy use to student outcomes or other measures. It is also suggested to investigate metamotivational processes in the field of faculty members in future studies.

## Supplementary Information

**Additional file 1: Appendix 1.** CVI and CVR index values.

**Additional file 2: Appendix 2.** Investigation of the validity evidence and reliability in seven models.

**Additional file 3: Appendix 3.** Structural model of predictive relationship between motivational regulation strategies and motivational components (The numbers inside ellipses are the R^2^ values).

## Data Availability

The datasets used and/or analysed during the current study are available from the corresponding author on reasonable request.
