# Supplementary material for: The role of motivational components in metamotivational monitoring in medical students: a mixed method study
Source: BMC Med Educ. 2023 Feb 13;23:108. doi: 10.1186/s12909-023-04081-y (PMC9924879; doi:10.1186/s12909-023-04081-y)
Supplement: Supplementary file 1 — Additional file 1: Appendix 1. CVI and CVR index values. [file 12909_2023_4081_MOESM1_ESM.docx]

Additional file 1: CVI and CVR index values

| Item number | CVI | | | Item-CVI | CVR  (N= 15, CVR_thresh_. = 0.49) | |
| --- | --- | --- | --- | --- | --- | --- |
|  | Relevance | Clarity | Simplicity |  |  |  |
| 1 | 93.33 | 100 | 100 | 97.78 | 1 | Accepted |
| 2 | 93.33 | 100 | 86.67 | 93.33 | 1 | Accepted |
| 3 | 100 | 93.33 | 93.33 | 95.55 | 0.87 | Accepted |
| 4 | 80 | 93.33 | 93.33 | 88.89 | 0.87 | Accepted |
| 5 | 100 | 93.33 | 86.67 | 93.33 | 0.87 | Accepted |
| 6 | 100 | 100 | 93.33 | 97.78 | 0.73 | Accepted |
| 7 | 100 | 100 | 86.67 | 95.56 | 1 | Accepted |
| 8 | 100 | 93.33 | 100 | 97.78 | 1 | Accepted |
| 9 | 86.67 | 86.67 | 100 | 91.11 | 1 | Accepted |
| 10 | 93.33 | 100 | 100 | 97.78 | 0.73 | Accepted |
| 11 | 100 | 100 | 86.67 | 95.56 | 0.87 | Accepted |
| 12 | 86.67 | 100 | 93.33 | 93.33 | 0.87 | Accepted |
| 13 | 80 | 86.67 | 100 | 88.89 | 1 | Accepted |
| 14 | 93.33 | 93.33 | 100 | 95.55 | 0.87 | Accepted |
| 15 | 100 | 100 | 93.33 | 97.78 | 0.87 | Accepted |
| 16 | 86.67 | 93.33 | 93.33 | 91.11 | 0.6 | Accepted |
| 17 | 100 | 93.33 | 93.33 | 95.55 | 0.73 | Accepted |
| 18 | 93.33 | 100 | 86.67 | 93.33 | 0.87 | Accepted |
| 19 | 86.67 | 100 | 100 | 95.56 | 1 | Accepted |
| 20 | 100 | 93.33 | 86.67 | 93.33 | 1 | Accepted |
| 21 | 86.67 | 100 | 100 | 95.56 | 0.73 | Accepted |
| 22 | 100 | 100 | 93.33 | 97.78 | 0.87 | Accepted |
| 23 | 93.33 | 86.67 | 86.67 | 88.89 | 0.87 | Accepted |
| 24 | 100 | 100 | 100 | 100 | 1 | Accepted |
| Average | 93.89 | 96.11 | 93.89 | 94.63 |  |  |
