# Supplementary material for: The role of motivational components in metamotivational monitoring in medical students: a mixed method study
Source: BMC Med Educ. 2023 Feb 13;23:108. doi: 10.1186/s12909-023-04081-y (PMC9924879; doi:10.1186/s12909-023-04081-y)
Supplement: Supplementary file 2 — Additional file 2: Appendix 2. Investigation of the validity evidence and reliability in seven models. [file 12909_2023_4081_MOESM2_ESM.docx]

Additional file 2: Investigation of the validity evidence and reliability in seven models

Table 1: Investigation of convergent validity and reliability of items in the structural model of the predictive relationship between regulation of value strategy and motivational components

|  | Item number | Factor loadings | T-value | AVE | Composite reliability | Cronbach's alpha |
| --- | --- | --- | --- | --- | --- | --- |
| Regulation of value | 1 | 0.79 | 16.03 | 0.61 | 0.89 | 0.84 |
|  | 2 | 0.83 | 20.79 |  |  |  |
|  | 3 | 0.72 | 11.18 |  |  |  |
|  | 4 | 0.68 | 12.08 |  |  |  |
|  | 5 | 0.86 | 32.39 |  |  |  |
| Promotion value | 1 | 0.88 | 35.38 | 0.81 | 0.94 | 0.92 |
|  | 2 | 0.91 | 30.28 |  |  |  |
|  | 3 | 0.94 | 57.33 |  |  |  |
|  | 4 | 0.87 | 20.97 |  |  |  |
| Prevention value | 1 | 0.87 | 14.09 | 0.75 | 0.92 | 0.89 |
|  | 2 | 0.79 | 6.58 |  |  |  |
|  | 3 | 0.90 | 11.92 |  |  |  |
|  | 4 | 0.91 | 18.43 |  |  |  |
| Self-efficacy | 1 | 0.73 | 9.97 | 0.65 | 0.88 | 0.82 |
|  | 2 | 0.84 | 22.97 |  |  |  |
|  | 3 | 0.87 | 32.94 |  |  |  |
|  | 4 | 0.78 | 16.18 |  |  |  |
| External value | 1 | 0.89 | 9.28 | 0.72 | 0.91 | 0.87 |
|  | 2 | 0.79 | 8.62 |  |  |  |
|  | 3 | 0.92 | 10.43 |  |  |  |
|  | 4 | 0.78 | 7.41 |  |  |  |
| Intrinsic value | 1 | 0.86 | 32.66 | 0.72 | 0.91 | 0.87 |
|  | 2 | 0.88 | 34.05 |  |  |  |
|  | 3 | 0.85 | 22.57 |  |  |  |
|  | 4 | 0.80 | 12.46 |  |  |  |
| Self-relevant value | 1 | 0.88 | 38.05 | 0.77 | 0.93 | 0.90 |
|  | 2 | 0.87 | 29.05 |  |  |  |
|  | 3 | 0.89 | 34.93 |  |  |  |
|  | 4 | 0.86 | 29.62 |  |  |  |

Table 2: Investigation of divergent validity of items in the structural model of the predictive relationship between regulation of value strategy and motivational components

|  | External value | Intrinsic value | Prevention value | Promotion value | Self-efficacy | Self-relevant value | Regulation of value |
| --- | --- | --- | --- | --- | --- | --- | --- |
| SEF1 | 0.30 | 0.37 | 0.20 | 0.33 | 0.73 | 0.34 | 0.40 |
| SEF2 | 0.44 | 0.62 | 0.45 | 0.62 | 0.84 | 0.49 | 0.55 |
| SEF3 | 0.32 | 0.51 | 0.40 | 0.46 | 0.87 | 0.48 | 0.59 |
| SEF4 | 0.50 | 0.53 | 0.44 | 0.49 | 0.78 | 0.41 | 0.49 |
| EXV1 | 0.89 | 0.48 | 0.42 | 0.61 | 0.50 | 0.33 | 0.26 |
| EXV2 | 0.79 | 0.31 | 0.21 | 0.44 | 0.36 | 0.36 | 0.21 |
| EXV3 | 0.92 | 0.46 | 0.32 | 0.53 | 0.42 | 0.33 | 0.26 |
| EXV4 | 0.78 | 0.38 | 0.38 | 0.55 | 0.32 | 0.34 | 0.20 |
| ITV1 | 0.37 | 0.86 | 0.43 | 0.60 | 0.62 | 0.65 | 0.73 |
| ITV2 | 0.38 | 0.88 | 0.48 | 0.62 | 0.58 | 0.55 | 0.59 |
| ITV3 | 0.41 | 0.85 | 0.29 | 0.57 | 0.40 | 0.49 | 0.59 |
| ITV4 | 0.53 | 0.80 | 0.26 | 0.66 | 0.55 | 0.42 | 0.48 |
| PREV1 | 0.33 | 0.40 | 0.87 | 0.55 | 0.43 | 0.40 | 0.29 |
| PREV2 | 0.36 | 0.25 | 0.79 | 0.47 | 0.43 | 0.22 | 0.17 |
| PREV3 | 0.34 | 0.31 | 0.90 | 0.51 | 0.29 | 0.27 | 0.21 |
| PREV4 | 0.35 | 0.49 | 0.91 | 0.68 | 0.47 | 0.36 | 0.35 |
| PROV1 | 0.44 | 0.65 | 0.66 | 0.88 | 0.49 | 0.55 | 0.56 |
| PROV2 | 0.61 | 0.63 | 0.51 | 0.91 | 0.56 | 0.50 | 0.45 |
| PROV3 | 0.68 | 0.69 | 0.62 | 0.94 | 0.58 | 0.49 | 0.48 |
| PROV4 | 0.55 | 0.61 | 0.55 | 0.87 | 0.54 | 0.40 | 0.38 |
| STRA1 | 0.09 | 0.55 | 0.26 | 0.30 | 0.53 | 0.53 | 0.79 |
| STRA2 | 0.34 | 0.59 | 0.23 | 0.45 | 0.55 | 0.65 | 0.83 |
| STRA3 | 0.07 | 0.47 | 0.03 | 0.29 | 0.40 | 0.45 | 0.72 |
| STRA4 | 0.29 | 0.49 | 0.30 | 0.49 | 0.49 | 0.54 | 0.68 |
| STRA5 | 0.23 | 0.68 | 0.35 | 0.48 | 0.51 | 0.64 | 0.86 |
| SRV1 | 0.31 | 0.58 | 0.36 | 0.44 | 0.48 | 0.88 | 0.62 |
| SRV2 | 0.22 | 0.55 | 0.36 | 0.46 | 0.44 | 0.87 | 0.66 |
| SRV3 | 0.41 | 0.54 | 0.38 | 0.52 | 0.51 | 0.89 | 0.65 |
| SRV4 | 0.44 | 0.56 | 0.23 | 0.50 | 0.45 | 0.86 | 0.61 |

Table 3: Investigation of convergent validity and reliability of items in the structural model of the predictive relationship between regulation of situational interest strategy and motivational components

|  | Item number | Factor loadings | T-value | AVE | Composite reliability | Cronbach's alpha |
| --- | --- | --- | --- | --- | --- | --- |
| Regulation of situational interest | 1 | 0.64 | 5.74 | 0.54 | 0.82 | 0.71 |
|  | 2 | 0.89 | 45.87 |  |  |  |
|  | 3 | 0.76 | 13.55 |  |  |  |
|  | 4 | 0.60 | 4.98 |  |  |  |
| Self-efficacy | 1 | 0.87 | 29.70 | 0.75 | 0.92 | 0.89 |
|  | 2 | 0.90 | 45.45 |  |  |  |
|  | 3 | 0.85 | 21.88 |  |  |  |
|  | 4 | 0.84 | 19.27 |  |  |  |
| External value | 1 | 0.88 | 22.35 | 0.75 | 0.92 | 0.89 |
|  | 2 | 0.86 | 25.94 |  |  |  |
|  | 3 | 0.89 | 35.46 |  |  |  |
|  | 4 | 0.83 | 16.75 |  |  |  |
| Intrinsic value | 1 | 0.84 | 25.41 | 0.70 | 0.90 | 0.86 |
|  | 2 | 0.91 | 49.30 |  |  |  |
|  | 3 | 0.76 | 12.29 |  |  |  |
|  | 4 | 0.82 | 17.23 |  |  |  |
| Prevention value | 1 | 0.81 | 15.03 | 0.66 | 0.89 | 0.83 |
|  | 2 | 0.83 | 12.62 |  |  |  |
|  | 3 | 0.90 | 25.21 |  |  |  |
|  | 4 | 0.71 | 7.77 |  |  |  |
| Promotion value | 1 | 0.92 | 52.44 | 0.84 | 0.96 | 0.94 |
|  | 2 | 0.94 | 59.71 |  |  |  |
|  | 3 | 0.91 | 29.80 |  |  |  |
|  | 4 | 0.90 | 36.01 |  |  |  |
| Self-relevant value | 1 | 0.85 | 24.04 | 0.69 | 0.90 | 0.85 |
|  | 2 | 0.82 | 18.38 |  |  |  |
|  | 3 | 0.76 | 11.01 |  |  |  |
|  | 4 | 0.89 | 36.47 |  |  |  |

Table 4: Investigation of divergent validity of items in the structural model of the predictive relationship between regulation of situational interest strategy and motivational components

|  | External value | Intrinsic value | Prevention value | Promotion value | Self-efficacy | Self-relevant value | Regulation of situational interest |
| --- | --- | --- | --- | --- | --- | --- | --- |
| SEF1 | 0.38 | 0.62 | 0.36 | 0.60 | 0.87 | 0.49 | 0.47 |
| SEF2 | 0.50 | 0.56 | 0.56 | 0.76 | 0.90 | 0.65 | 0.49 |
| SEF3 | 0.47 | 0.43 | 0.35 | 0.53 | 0.85 | 0.51 | 0.41 |
| SEF4 | 0.41 | 0.42 | 0.46 | 0.50 | 0.84 | 0.62 | 0.35 |
| EXV1 | 0.88 | 0.32 | 0.54 | 0.64 | 0.46 | 0.32 | 0.38 |
| EXV2 | 0.86 | 0.27 | 0.54 | 0.53 | 0.37 | 0.29 | 0.36 |
| EXV3 | 0.89 | 0.23 | 0.57 | 0.64 | 0.52 | 0.36 | 0.32 |
| EXV4 | 0.83 | 0.30 | 0.55 | 0.49 | 0.40 | 0.32 | 0.29 |
| ITV1 | 0.21 | 0.84 | 0.25 | 0.50 | 0.52 | 0.52 | 0.60 |
| ITV2 | 0.38 | 0.91 | 0.40 | 0.60 | 0.61 | 0.52 | 0.67 |
| ITV3 | 0.20 | 0.76 | 0.22 | 0.37 | 0.40 | 0.37 | 0.53 |
| ITV4 | 0.28 | 0.82 | 0.32 | 0.32 | 0.45 | 0.43 | 0.58 |
| PREV1 | 0.55 | 0.19 | 0.81 | 0.51 | 0.43 | 0.47 | 0.32 |
| PREV2 | 0.64 | 0.26 | 0.83 | 0.50 | 0.41 | 0.42 | 0.27 |
| PREV3 | 0.66 | 0.36 | 0.90 | 0.63 | 0.48 | 0.50 | 0.41 |
| PREV4 | 0.27 | 0.32 | 0.71 | 0.32 | 0.31 | 0.33 | 0.48 |
| PROV1 | 0.68 | 0.51 | 0.60 | 0.92 | 0.68 | 0.61 | 0.58 |
| PROV2 | 0.65 | 0.48 | 0.58 | 0.94 | 0.64 | 0.66 | 0.55 |
| PROV3 | 0.59 | 0.51 | 0.58 | 0.91 | 0.66 | 0.56 | 0.45 |
| PROV4 | 0.52 | 0.50 | 0.41 | 0.90 | 0.60 | 0.61 | 0.48 |
| STRA1 | 0.24 | 0.42 | 0.27 | 0.36 | 0.31 | 0.28 | 0.64 |
| STRA2 | 0.39 | 0.72 | 0.49 | 0.57 | 0.56 | 0.51 | 0.89 |
| STRA3 | 0.29 | 0.48 | 0.40 | 0.38 | 0.28 | 0.35 | 0.76 |
| STRA4 | 0.17 | 0.40 | 0.15 | 0.25 | 0.22 | 0.26 | 0.60 |
| SRV1 | 0.30 | 0.50 | 0.55 | 0.55 | 0.57 | 0.85 | 0.42 |
| SRV2 | 0.48 | 0.53 | 0.42 | 0.65 | 0.52 | 0.82 | 0.46 |
| SRV3 | 0.17 | 0.33 | 0.34 | 0.43 | 0.47 | 0.76 | 0.35 |
| SRV4 | 0.25 | 0.46 | 0.44 | 0.56 | 0.60 | 0.89 | 0.42 |

Table 5: Investigation of convergent validity and reliability of items in the structural model of the predictive relationship between regulation of relatedness strategy and motivational components

|  | Item number | Factor loadings | T-value | AVE | Composite reliability | Cronbach's alpha |
| --- | --- | --- | --- | --- | --- | --- |
| Regulation of relatedness | 1 | 0.64 | 6.11 | 0.49 | 0.74 | 0.55 |
|  | 2 | 0.58 | 3.60 |  |  |  |
|  | 3 | 0.70 | 5.95 |  |  |  |
|  | 4 | 0.64 | 5.52 |  |  |  |
| Self-efficacy | 1 | 0.91 | 43.03 | 0.80 | 0.94 | 0.92 |
|  | 2 | 0.95 | 82.94 |  |  |  |
|  | 3 | 0.84 | 13.85 |  |  |  |
|  | 4 | 0.89 | 26.77 |  |  |  |
| External value | 1 | 0.88 | 5.71 | 0.51 | 0.80 | 0.76 |
|  | 2 | 0.68 | 3.25 |  |  |  |
|  | 3 | 0.76 | 3.12 |  |  |  |
|  | 4 | 0.48 | 2.29 |  |  |  |
| Intrinsic value | 1 | 0.93 | 50.33 | 0.79 | 0.94 | 0.91 |
|  | 2 | 0.95 | 101.91 |  |  |  |
|  | 3 | 0.77 | 7.33 |  |  |  |
|  | 4 | 0.89 | 17.58 |  |  |  |
| Prevention value | 1 | 0.90 | 26.96 | 0.75 | 0.92 | 0.89 |
|  | 2 | 0.87 | 18.55 |  |  |  |
|  | 3 | 0.96 | 57.99 |  |  |  |
|  | 4 | 0.73 | 7.98 |  |  |  |
| Promotion value | 1 | 0.91 | 38.26 | 0.87 | 0.97 | 0.95 |
|  | 2 | 0.94 | 56.52 |  |  |  |
|  | 3 | 0.95 | 88.59 |  |  |  |
|  | 4 | 0.94 | 66.22 |  |  |  |
| Self-relevant value | 1 | 0.88 | 32.48 | 0.79 | 0.99 | 0.91 |
|  | 2 | 0.93 | 42.06 |  |  |  |
|  | 3 | 0.82 | 13.64 |  |  |  |
|  | 4 | 0.92 | 46.48 |  |  |  |

Table 6: Investigation of divergent validity of items in the structural model of the predictive relationship between regulation of regulation of relatedness strategy and motivational components

|  | External value | Intrinsic value | Prevention value | Promotion value | Self-efficacy | Self-relevant value | **R**egulation of relatedness |
| --- | --- | --- | --- | --- | --- | --- | --- |
| SEF1 | 0.42 | 0.55 | 0.39 | 0.57 | 0.91 | 0.57 | 0.34 |
| SEF2 | 0.52 | 0.58 | 0.49 | 0.75 | 0.95 | 0.66 | 0.45 |
| SEF3 | 0.37 | 0.32 | 0.41 | 0.55 | 0.84 | 0.36 | 0.20 |
| SEF4 | 0.40 | 0.64 | 0.41 | 0.63 | 0.89 | 0.49 | 0.26 |
| EXV1 | 0.88 | 0.21 | 0.43 | 0.45 | 0.46 | 0.33 | 0.30 |
| EXV2 | 0.68 | 0.20 | 0.30 | 0.27 | 0.30 | 0.31 | 0.18 |
| EXV3 | 0.76 | 0.17 | 0.28 | 0.32 | 0.34 | 0.23 | 0.09 |
| EXV4 | 0.48 | 0.24 | 0.24 | 0.16 | 0.29 | 0.08 | 0.00 |
| ITV1 | 0.21 | 0.93 | 0.34 | 0.66 | 0.52 | 0.59 | 0.47 |
| ITV2 | 0.28 | 0.95 | 0.33 | 0.60 | 0.56 | 0.61 | 0.46 |
| ITV3 | 0.16 | 0.77 | 0.23 | 0.37 | 0.48 | 0.41 | 0.20 |
| ITV4 | 0.22 | 0.89 | 0.29 | 0.53 | 0.59 | 0.52 | 0.28 |
| PREV1 | 0.39 | 0.30 | 0.90 | 0.47 | 0.44 | 0.31 | 0.31 |
| PREV2 | 0.48 | 0.27 | 0.87 | 0.36 | 0.38 | 0.36 | 0.27 |
| PREV3 | 0.49 | 0.34 | 0.96 | 0.57 | 0.50 | 0.46 | 0.37 |
| PREV4 | 0.16 | 0.28 | 0.73 | 0.31 | 0.31 | 0.19 | 0.18 |
| PROV1 | 0.42 | 0.59 | 0.48 | 0.91 | 0.71 | 0.55 | 0.51 |
| PROV2 | 0.42 | 0.58 | 0.41 | 0.94 | 0.72 | 0.66 | 0.50 |
| PROV3 | 0.52 | 0.59 | 0.52 | 0.95 | 0.62 | 0.72 | 0.50 |
| PROV4 | 0.40 | 0.60 | 0.50 | 0.94 | 0.61 | 0.70 | 0.50 |
| STRA1 | 0.16 | 0.37 | 0.09 | 0.41 | 0.22 | 0.54 | 0.64 |
| STRA2 | 0.07 | 0.04 | 0.12 | 0.16 | 0.20 | 0.19 | 0.58 |
| STRA3 | 0.30 | 0.19 | 0.27 | 0.39 | 0.20 | 0.24 | 0.70 |
| STRA4 | 0.16 | 0.37 | 0.35 | 0.33 | 0.33 | 0.26 | 0.64 |
| SRV1 | 0.35 | 0.56 | 0.29 | 0.62 | 0.50 | 0.88 | 0.46 |
| SRV2 | 0.36 | 0.63 | 0.40 | 0.68 | 0.65 | 0.93 | 0.55 |
| SRV3 | 0.31 | 0.44 | 0.35 | 0.48 | 0.46 | 0.82 | 0.33 |
| SRV4 | 0.35 | 0.52 | 0.38 | 0.69 | 0.53 | 0.92 | 0.45 |

Table 7: Investigation of convergent validity and reliability of items in the structural model of the predictive relationship between promotional situational awareness strategy and motivational components

|  | Item number | Factor loadings | T-value | AVE | Composite reliability | Cronbach's alpha |
| --- | --- | --- | --- | --- | --- | --- |
| Promotional situational awareness | 1 | 0.64 | 8.39 | 0.58 | 0.85 | 0.76 |
|  | 2 | 0.74 | 14.55 |  |  |  |
|  | 3 | 0.88 | 23.43 |  |  |  |
|  | 4 | 0.78 | 13.17 |  |  |  |
| Self-efficacy | 1 | 0.79 | 14.78 | 0.74 | 0.92 | 0.88 |
|  | 2 | 0.88 | 23.43 |  |  |  |
|  | 3 | 0.89 | 31.47 |  |  |  |
|  | 4 | 0.87 | 24.63 |  |  |  |
| External value | 1 | 0.74 | 9.68 | 0.62 | 0.87 | 0.80 |
|  | 2 | 0.74 | 12.42 |  |  |  |
|  | 3 | 0.87 | 25.54 |  |  |  |
|  | 4 | 0.79 | 19.20 |  |  |  |
| Intrinsic value | 1 | 0.86 | 20.36 | 0.65 | 0.88 | 0.82 |
|  | 2 | 0.89 | 29.15 |  |  |  |
|  | 3 | 0.78 | 9.89 |  |  |  |
|  | 4 | 0.68 | 5.63 |  |  |  |
| Prevention value | 1 | 0.82 | 8.95 | 0.56 | 0.83 | 0.72 |
|  | 2 | 0.80 | 12.48 |  |  |  |
|  | 3 | 0.86 | 9.81 |  |  |  |
|  | 4 | 0.44 | 4.19 |  |  |  |
| Promotion value | 1 | 0.86 | 21.88 | 0.77 | 0.93 | 0.90 |
|  | 2 | 0.91 | 24.14 |  |  |  |
|  | 3 | 0.93 | 54.60 |  |  |  |
|  | 4 | 0.80 | 16.45 |  |  |  |
| Self-relevant value | 1 | 0.83 | 20.70 | 0.78 | 0.93 | 0.91 |
|  | 2 | 0.92 | 43.77 |  |  |  |
|  | 3 | 0.86 | 21.93 |  |  |  |
|  | 4 | 0.93 | 51.15 |  |  |  |

Table 8: Investigation of divergent validity of items in the structural model of the predictive relationship between regulation of promotional situational awareness strategy and motivational components

|  | External value | Intrinsic value | Prevention value | Promotion value | Self-efficacy | Self-relevant value | Promotional situational awareness |
| --- | --- | --- | --- | --- | --- | --- | --- |
| SEF1 | 0.03 | 0.57 | 0.26 | 0.48 | 0.79 | 0.54 | 0.42 |
| SEF2 | 0.09 | 0.56 | 0.25 | 0.67 | 0.88 | 0.65 | 0.53 |
| SEF3 | 0.05 | 0.57 | 0.25 | 0.49 | 0.89 | 0.62 | 0.49 |
| SEF4 | 0.16 | 0.58 | 0.27 | 0.64 | 0.87 | 0.78 | 0.54 |
| EXV1 | 0.74 | -0.08 | 0.34 | 0.38 | 0.07 | 0.14 | 0.33 |
| EXV2 | 0.74 | 0.03 | 0.38 | 0.13 | -0.03 | 0.11 | 0.25 |
| EXV3 | 0.87 | 0.05 | 0.32 | 0.39 | 0.09 | 0.24 | 0.44 |
| EXV4 | 0.79 | 0.09 | 0.45 | 0.15 | 0.06 | 0.12 | 0.32 |
| ITV1 | 0.01 | 0.86 | 0.14 | 0.31 | 0.60 | 0.58 | 0.38 |
| ITV2 | 0.00 | 0.89 | 0.09 | 0.27 | 0.55 | 0.58 | 0.37 |
| ITV3 | 0.11 | 0.78 | 0.09 | 0.21 | 0.49 | 0.52 | 0.25 |
| ITV4 | 0.01 | 0.68 | 0.08 | 0.28 | 0.48 | 0.32 | 0.28 |
| PREV1 | 0.30 | -0.01 | 0.82 | 0.40 | 0.19 | 0.17 | 0.21 |
| PREV2 | 0.48 | 0.09 | 0.80 | 0.41 | 0.26 | 0.26 | 0.42 |
| PREV3 | 0.40 | 0.05 | 0.86 | 0.40 | 0.17 | 0.17 | 0.28 |
| PREV4 | 0.07 | 0.23 | 0.44 | 0.30 | 0.23 | 0.34 | 0.24 |
| PROV1 | 0.24 | 0.38 | 0.42 | 0.86 | 0.57 | 0.60 | 0.55 |
| PROV2 | 0.25 | 0.35 | 0.39 | 0.91 | 0.66 | 0.61 | 0.57 |
| PROV3 | 0.42 | 0.27 | 0.53 | 0.93 | 0.57 | 0.51 | 0.64 |
| PROV4 | 0.31 | 0.20 | 0.46 | 0.80 | 0.56 | 0.47 | 0.54 |
| STRA1 | 0.38 | 0.26 | 0.41 | 0.35 | 0.37 | 0.26 | 0.64 |
| STRA2 | 0.45 | 0.39 | 0.31 | 0.55 | 0.43 | 0.37 | 0.74 |
| STRA3 | 0.25 | 0.28 | 0.26 | 0.60 | 0.51 | 0.49 | 0.88 |
| STRA4 | 0.26 | 0.29 | 0.32 | 0.48 | 0.43 | 0.42 | 0.78 |
| SRV1 | 0.22 | 0.51 | 0.40 | 0.55 | 0.68 | 0.83 | 0.48 |
| SRV2 | 0.24 | 0.55 | 0.27 | 0.66 | 0.71 | 0.92 | 0.47 |
| SRV3 | 0.11 | 0.53 | 0.23 | 0.47 | 0.58 | 0.86 | 0.40 |
| SRV4 | 0.13 | 0.63 | 0.22 | 0.51 | 0.70 | 0.93 | 0.45 |

Table 9: Investigation of convergent validity and reliability of items in the structural model of the predictive relationship between preventional situational awareness strategy and motivational components

|  | Item number | Factor loadings | T-value | AVE | Composite reliability | Cronbach's alpha |
| --- | --- | --- | --- | --- | --- | --- |
| Preventional situational awareness | 1 | 0.57 | 3.98 | 0.51 | 0.80 | 0.70 |
|  | 2 | 0.67 | 5.07 |  |  |  |
|  | 3 | 0.81 | 15.45 |  |  |  |
|  | 4 | 0.79 | 11.80 |  |  |  |
| Self-efficacy | 1 | 0.80 | 10.51 | 0.70 | 0.91 | 0.86 |
|  | 2 | 0.85 | 14.56 |  |  |  |
|  | 3 | 0.84 | 12.48 |  |  |  |
|  | 4 | 0.86 | 11.01 |  |  |  |
| External value | 1 | 0.50 | 3.70 | 0.50 | 0.79 | 0.65 |
|  | 2 | 0.58 | 4.29 |  |  |  |
|  | 3 | 0.84 | 18.35 |  |  |  |
|  | 4 | 0.83 | 14.11 |  |  |  |
| Intrinsic value | 1 | 0.88 | 6.92 | 0.70 | 0.90 | 0.86 |
|  | 2 | 0.93 | 8.40 |  |  |  |
|  | 3 | 0.75 | 5.01 |  |  |  |
|  | 4 | 0.76 | 5.66 |  |  |  |
| Prevention value | 1 | 0.79 | 11.19 | 0.65 | 0.88 | 0.82 |
|  | 2 | 0.84 | 17.76 |  |  |  |
|  | 3 | 0.92 | 31.51 |  |  |  |
|  | 4 | 0.64 | 5.65 |  |  |  |
| Promotion value | 1 | 0.88 | 24.71 | 0.79 | 0.94 | 0.91 |
|  | 2 | 0.90 | 31.02 |  |  |  |
|  | 3 | 0.93 | 41.98 |  |  |  |
|  | 4 | 0.85 | 17.20 |  |  |  |
| Self-relevant value | 1 | 0.81 | 6.11 | 0.72 | 0.91 | 0.87 |
|  | 2 | 0.89 | 7.89 |  |  |  |
|  | 3 | 0.88 | 10.03 |  |  |  |
|  | 4 | 0.82 | 10.11 |  |  |  |

Table 10: Investigation of divergent validity of items in the structural model of the predictive relationship between regulation of preventional situational awareness strategy and motivational components

|  | External value | Intrinsic value | Prevention value | Promotion value | Self-efficacy | Self-relevant value | Preventional situational awareness |
| --- | --- | --- | --- | --- | --- | --- | --- |
| SEF1 | 0.15 | 0.35 | 0.28 | 0.36 | 0.80 | 0.42 | 0.26 |
| SEF2 | 0.31 | 0.51 | 0.43 | 0.71 | 0.85 | 0.77 | 0.29 |
| SEF3 | 0.25 | 0.34 | 0.25 | 0.44 | 0.84 | 0.49 | 0.30 |
| SEF4 | 0.36 | 0.56 | 0.34 | 0.64 | 0.86 | 0.65 | 0.30 |
| EXV1 | 0.50 | 0.12 | 0.35 | 0.31 | 0.28 | 0.40 | 0.25 |
| EXV2 | 0.58 | 0.12 | 0.18 | 0.10 | 0.14 | 0.09 | 0.20 |
| EXV3 | 0.84 | 0.15 | 0.37 | 0.34 | 0.29 | 0.26 | 0.40 |
| EXV4 | 0.83 | 0.14 | 0.35 | 0.29 | 0.20 | 0.19 | 0.42 |
| ITV1 | 0.12 | 0.88 | 0.14 | 0.44 | 0.45 | 0.49 | 0.13 |
| ITV2 | 0.16 | 0.93 | 0.19 | 0.57 | 0.43 | 0.58 | 0.21 |
| ITV3 | 0.22 | 0.75 | 0.17 | 0.42 | 0.48 | 0.50 | 0.09 |
| ITV4 | 0.17 | 0.76 | 0.26 | 0.53 | 0.47 | 0.53 | 0.09 |
| PREV1 | 0.28 | 0.29 | 0.79 | 0.46 | 0.35 | 0.53 | 0.26 |
| PREV2 | 0.38 | 0.04 | 0.84 | 0.28 | 0.29 | 0.31 | 0.40 |
| PREV3 | 0.46 | 0.17 | 0.92 | 0.41 | 0.27 | 0.40 | 0.38 |
| PREV4 | 0.31 | 0.33 | 0.64 | 0.53 | 0.43 | 0.52 | 0.20 |
| PROV1 | 0.24 | 0.54 | 0.41 | 0.88 | 0.55 | 0.69 | 0.27 |
| PROV2 | 0.39 | 0.53 | 0.38 | 0.90 | 0.60 | 0.68 | 0.31 |
| PROV3 | 0.39 | 0.54 | 0.48 | 0.93 | 0.60 | 0.71 | 0.29 |
| PROV4 | 0.34 | 0.49 | 0.47 | 0.85 | 0.55 | 0.66 | 0.23 |
| STRA1 | 0.10 | -0.02 | 0.20 | 0.08 | 0.12 | 0.01 | 0.57 |
| STRA2 | 0.18 | 0.26 | 0.25 | 0.25 | 0.28 | 0.30 | 0.67 |
| STRA3 | 0.32 | 0.12 | 0.27 | 0.20 | 0.22 | 0.17 | 0.81 |
| STRA4 | 0.57 | 0.08 | 0.38 | 0.28 | 0.30 | 0.15 | 0.79 |
| SRV1 | 0.26 | 0.61 | 0.40 | 0.72 | 0.69 | 0.81 | 0.15 |
| SRV2 | 0.29 | 0.58 | 0.48 | 0.68 | 0.58 | 0.89 | 0.18 |
| SRV3 | 0.38 | 0.49 | 0.48 | 0.73 | 0.60 | 0.88 | 0.23 |
| SRV4 | 0.16 | 0.50 | 0.37 | 0.52 | 0.54 | 0.82 | 0.24 |

Table 11: Investigation of convergent validity and reliability of items in the structural model of the predictive relationship between environmental structuring strategy and motivational components

|  | Item number | Factor loadings | T-value | AVE | Composite reliability | Cronbach's alpha |
| --- | --- | --- | --- | --- | --- | --- |
| Environmental structuring | 1 | 0.82 | 16.44 | 0.59 | 0.85 | 0.77 |
|  | 2 | 0.77 | 15.46 |  |  |  |
|  | 3 | 0.81 | 16.00 |  |  |  |
|  | 4 | 0.65 | 5.66 |  |  |  |
| Self-efficacy | 1 | 0.75 | 7.11 | 0.70 | 0.90 | 0.86 |
|  | 2 | 0.87 | 19.87 |  |  |  |
|  | 3 | 0.94 | 49.58 |  |  |  |
|  | 4 | 0.77 | 8.87 |  |  |  |
| External value | 1 | 0.53 | 2.88 | 0.64 | 0.87 | 0.81 |
|  | 2 | 0.80 | 8.09 |  |  |  |
|  | 3 | 0.95 | 8.81 |  |  |  |
|  | 4 | 0.87 | 6.97 |  |  |  |
| Intrinsic value | 1 | 0.81 | 14.23 | 0.72 | 0.91 | 0.88 |
|  | 2 | 0.90 | 39.77 |  |  |  |
|  | 3 | 0.83 | 9.55 |  |  |  |
|  | 4 | 0.85 | 18.85 |  |  |  |
| Prevention value | 1 | 0.84 | 8.97 | 0.73 | 0.89 | 0.83 |
|  | 2 | 0.74 | 5.50 |  |  |  |
|  | 3 | 0.96 | 35.75 |  |  |  |
| Promotion value | 1 | 0.91 | 39.95 | 0.80 | 0.94 | 0.92 |
|  | 2 | 0.89 | 37.06 |  |  |  |
|  | 3 | 0.93 | 38.43 |  |  |  |
|  | 4 | 0.85 | 27.40 |  |  |  |
| Self-relevant value | 1 | 0.88 | 23.89 | 0.80 | 0.94 | 0.92 |
|  | 2 | 0.92 | 50.29 |  |  |  |
|  | 3 | 0.89 | 36.85 |  |  |  |
|  | 4 | 0.89 | 37.61 |  |  |  |

Table 12: Investigation of divergent validity of items in the structural model of the predictive relationship between regulation of environmental structuring strategy and motivational components

|  | External value | Intrinsic value | Prevention value | Promotion value | Self-efficacy | Self-relevant value | Environmental structuring |
| --- | --- | --- | --- | --- | --- | --- | --- |
| SEF1 | 0.28 | 0.30 | 0.24 | 0.45 | 0.75 | 0.48 | 0.22 |
| SEF2 | 0.33 | 0.42 | 0.42 | 0.61 | 0.87 | 0.52 | 0.35 |
| SEF3 | 0.39 | 0.34 | 0.40 | 0.60 | 0.94 | 0.56 | 0.36 |
| SEF4 | 0.28 | 0.34 | 0.24 | 0.55 | 0.77 | 0.51 | 0.14 |
| EXV1 | 0.53 | 0.17 | 0.22 | 0.48 | 0.32 | 0.29 | 0.11 |
| EXV2 | 0.80 | 0.11 | 0.23 | 0.34 | 0.11 | 0.27 | 0.18 |
| EXV3 | 0.95 | 0.29 | 0.38 | 0.48 | 0.38 | 0.42 | 0.28 |
| EXV4 | 0.87 | 0.20 | 0.33 | 0.40 | 0.42 | 0.45 | 0.27 |
| ITV1 | 0.11 | 0.81 | 0.10 | 0.21 | 0.26 | 0.55 | 0.29 |
| ITV2 | 0.22 | 0.90 | 0.37 | 0.35 | 0.40 | 0.64 | 0.55 |
| ITV3 | 0.21 | 0.83 | 0.18 | 0.38 | 0.30 | 0.57 | 0.33 |
| ITV4 | 0.27 | 0.85 | 0.29 | 0.26 | 0.42 | 0.57 | 0.45 |
| PREV1 | 0.20 | 0.17 | 0.84 | 0.52 | 0.39 | 0.33 | 0.25 |
| PREV2 | 0.37 | 0.19 | 0.74 | 0.40 | 0.27 | 0.28 | 0.11 |
| PREV3 | 0.39 | 0.34 | 0.96 | 0.50 | 0.38 | 0.41 | 0.48 |
| PROV1 | 0.38 | 0.33 | 0.51 | 0.91 | 0.63 | 0.72 | 0.40 |
| PROV2 | 0.49 | 0.34 | 0.51 | 0.89 | 0.67 | 0.61 | 0.39 |
| PROV3 | 0.47 | 0.32 | 0.42 | 0.93 | 0.52 | 0.66 | 0.29 |
| PROV4 | 0.49 | 0.26 | 0.50 | 0.85 | 0.50 | 0.65 | 0.29 |
| STRA1 | 0.32 | 0.44 | 0.38 | 0.30 | 0.35 | 0.43 | 0.82 |
| STRA2 | 0.19 | 0.25 | 0.45 | 0.38 | 0.21 | 0.34 | 0.77 |
| STRA3 | 0.25 | 0.48 | 0.28 | 0.33 | 0.28 | 0.55 | 0.81 |
| STRA4 | 0.05 | 0.35 | 0.09 | 0.18 | 0.21 | 0.40 | 0.65 |
| SRV1 | 0.38 | 0.57 | 0.38 | 0.68 | 0.54 | 0.88 | 0.42 |
| SRV2 | 0.45 | 0.64 | 0.39 | 0.70 | 0.57 | 0.92 | 0.55 |
| SRV3 | 0.43 | 0.58 | 0.32 | 0.60 | 0.53 | 0.89 | 0.56 |
| SRV4 | 0.34 | 0.66 | 0.39 | 0.68 | 0.56 | 0.89 | 0.47 |

Table 13: Investigation of convergent validity and reliability of items in the structural model of the predictive relationship between self-consequating strategy and motivational components

|  | Item number | Factor loadings | T-value | AVE | Composite reliability | Cronbach's alpha |
| --- | --- | --- | --- | --- | --- | --- |
| Self-consequating | 1 | 0.77 | 10.19 | 0.62 | 0.83 | 0.71 |
|  | 2 | 0.77 | 13.04 |  |  |  |
|  | 3 | 0.82 | 17.57 |  |  |  |
| Self-efficacy | 1 | 0.90 | 30.12 | 0.78 | 0.93 | 0.91 |
|  | 2 | 0.90 | 27.47 |  |  |  |
|  | 3 | 0.93 | 28.99 |  |  |  |
|  | 4 | 0.81 | 12.94 |  |  |  |
| External value | 1 | 0.75 | 11.99 | 0.68 | 0.89 | 0.84 |
|  | 2 | 0.87 | 19.33 |  |  |  |
|  | 3 | 0.86 | 23.06 |  |  |  |
|  | 4 | 0.81 | 15.01 |  |  |  |
| Intrinsic value | 1 | 0.85 | 28.09 | 0.76 | 0.93 | 0.89 |
|  | 2 | 0.90 | 26.06 |  |  |  |
|  | 3 | 0.88 | 30.77 |  |  |  |
|  | 4 | 0.85 | 27.08 |  |  |  |
| Prevention value | 1 | 0.88 | 29.62 | 0.65 | 0.88 | 0.82 |
|  | 2 | 0.69 | 7.00 |  |  |  |
|  | 3 | 0.88 | 12.46 |  |  |  |
|  | 4 | 0.76 | 11.28 |  |  |  |
| Promotion value | 1 | 0.89 | 29.86 | 0.82 | 0.95 | 0.93 |
|  | 2 | 0.90 | 33.47 |  |  |  |
|  | 3 | 0.95 | 120.33 |  |  |  |
|  | 4 | 0.88 | 36.05 |  |  |  |
| Self-relevant value | 1 | 0.94 | 69.42 | 0.87 | 0.96 | 0.95 |
|  | 2 | 0.94 | 52.13 |  |  |  |
|  | 3 | 0.93 | 49.76 |  |  |  |
|  | 4 | 0.92 | 41.50 |  |  |  |

Table 14: Investigation of divergent validity of items in the structural model of the predictive relationship between regulation of self-consequating strategy and motivational components

|  | External value | Intrinsic value | Prevention value | Promotion value | Self-efficacy | Self-relevant value | Self-consequating |
| --- | --- | --- | --- | --- | --- | --- | --- |
| SEF1 | 0.35 | 0.45 | 0.43 | 0.60 | 0.90 | 0.54 | 0.37 |
| SEF2 | 0.55 | 0.67 | 0.60 | 0.82 | 0.90 | 0.70 | 0.39 |
| SEF3 | 0.40 | 0.56 | 0.51 | 0.70 | 0.93 | 0.60 | 0.21 |
| SEF4 | 0.42 | 0.54 | 0.51 | 0.63 | 0.81 | 0.65 | 0.28 |
| EXV1 | 0.75 | 0.49 | 0.62 | 0.67 | 0.43 | 0.48 | 0.29 |
| EXV2 | 0.87 | 0.52 | 0.45 | 0.57 | 0.41 | 0.40 | 0.38 |
| EXV3 | 0.86 | 0.55 | 0.50 | 0.70 | 0.51 | 0.46 | 0.34 |
| EXV4 | 0.81 | 0.37 | 0.44 | 0.40 | 0.26 | 0.38 | 0.30 |
| ITV1 | 0.48 | 0.85 | 0.43 | 0.61 | 0.56 | 0.68 | 0.45 |
| ITV2 | 0.58 | 0.90 | 0.56 | 0.67 | 0.59 | 0.69 | 0.33 |
| ITV3 | 0.54 | 0.88 | 0.56 | 0.62 | 0.55 | 0.61 | 0.37 |
| ITV4 | 0.47 | 0.85 | 0.48 | 0.63 | 0.50 | 0.66 | 0.45 |
| PREV1 | 0.48 | 0.48 | 0.88 | 0.58 | 0.46 | 0.59 | 0.34 |
| PREV2 | 0.56 | 0.35 | 0.69 | 0.45 | 0.31 | 0.29 | 0.25 |
| PREV3 | 0.46 | 0.44 | 0.88 | 0.57 | 0.53 | 0.58 | 0.36 |
| PREV4 | 0.51 | 0.64 | 0.76 | 0.63 | 0.62 | 0.60 | 0.21 |
| PROV1 | 0.60 | 0.63 | 0.62 | 0.89 | 0.65 | 0.68 | 0.37 |
| PROV2 | 0.68 | 0.60 | 0.56 | 0.90 | 0.69 | 0.64 | 0.39 |
| PROV3 | 0.66 | 0.71 | 0.61 | 0.95 | 0.77 | 0.69 | 0.39 |
| PROV4 | 0.62 | 0.69 | 0.68 | 0.88 | 0.72 | 0.71 | 0.39 |
| STRA1 | 0.14 | 0.27 | 0.21 | 0.21 | 0.17 | 0.31 | 0.77 |
| STRA2 | 0.26 | 0.47 | 0.26 | 0.37 | 0.31 | 0.25 | 0.77 |
| STRA3 | 0.46 | 0.34 | 0.37 | 0.38 | 0.36 | 0.48 | 0.82 |
| SRV1 | 0.54 | 0.73 | 0.58 | 0.70 | 0.61 | 0.94 | 0.48 |
| SRV2 | 0.43 | 0.68 | 0.55 | 0.63 | 0.65 | 0.94 | 0.38 |
| SRV3 | 0.47 | 0.72 | 0.64 | 0.74 | 0.71 | 0.93 | 0.45 |
| SRV4 | 0.50 | 0.68 | 0.62 | 0.74 | 0.67 | 0.92 | 0.37 |
