# Supplementary material for: The role of motivational components in metamotivational monitoring in medical students: a mixed method study
Source: BMC Med Educ. 2023 Feb 13;23:108. doi: 10.1186/s12909-023-04081-y (PMC9924879; doi:10.1186/s12909-023-04081-y)
Supplement: Supplementary file 3 — Additional file 3: Appendix 3. Structural model of predictive relationship between motivational regulation strategies and motivational components (The numbers inside ellipses are the R2 values). [file 12909_2023_4081_MOESM3_ESM.docx]

Additional file 3: Structural model of predictive relationship between motivational regulation strategies and motivational components  **(**The numbers inside ellipses are the R^2^ values**)**


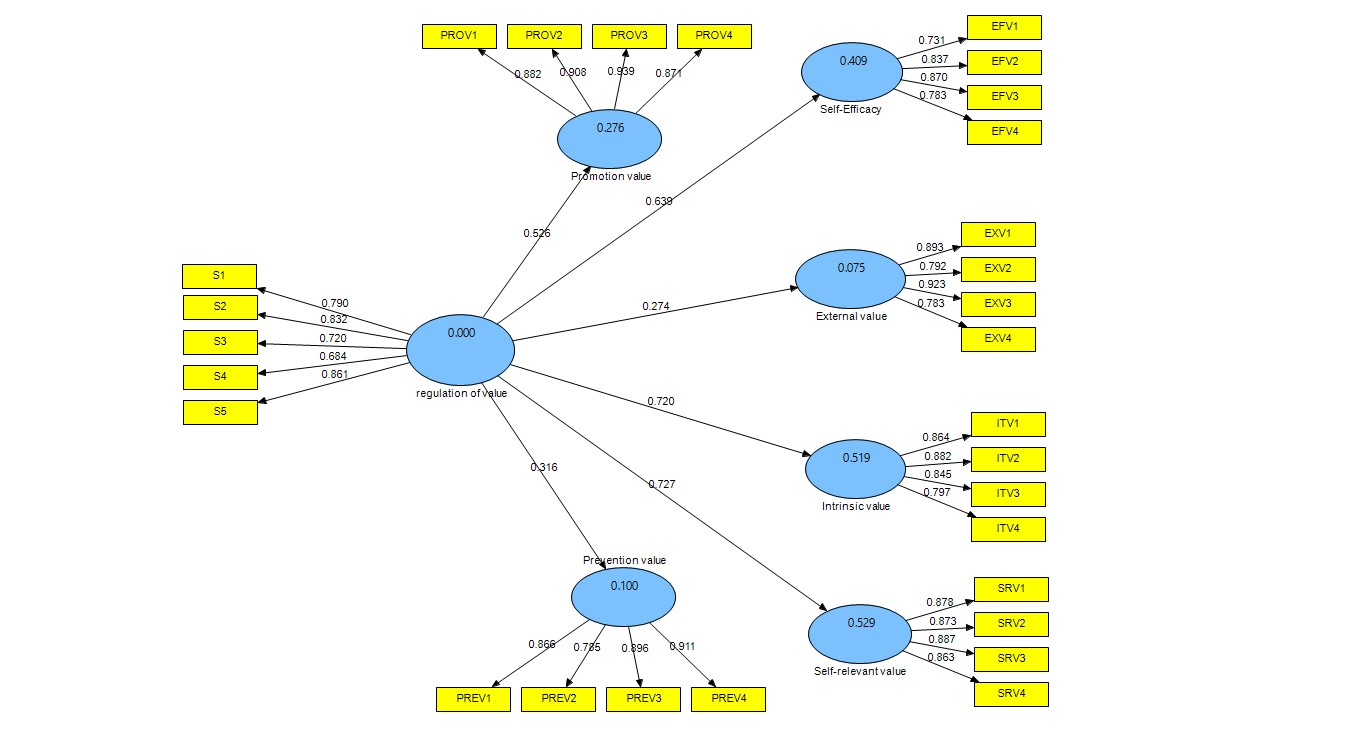


Figure 1: Structural model and predictive relationship between regulation of value strategy and motivational components


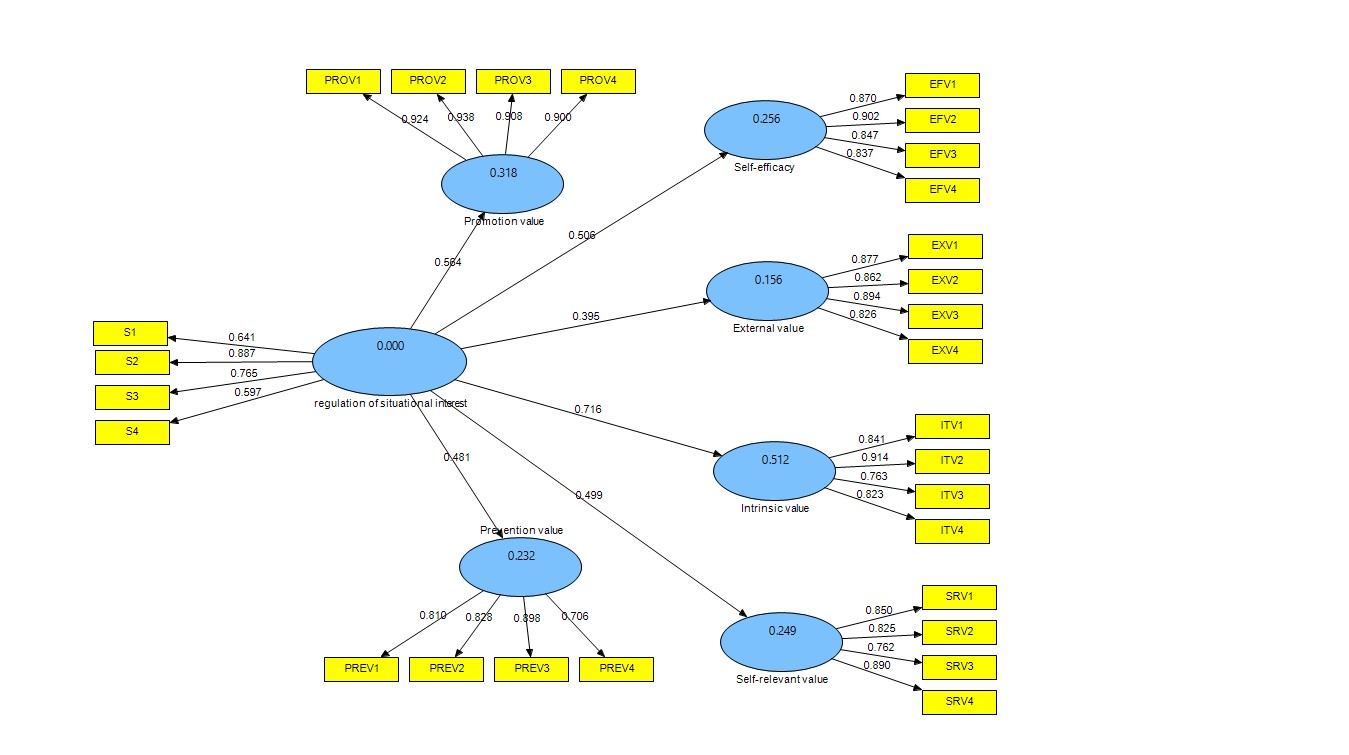


Figure 2: Structural model and predictive relationship between regulation of situational interest strategy and motivational components


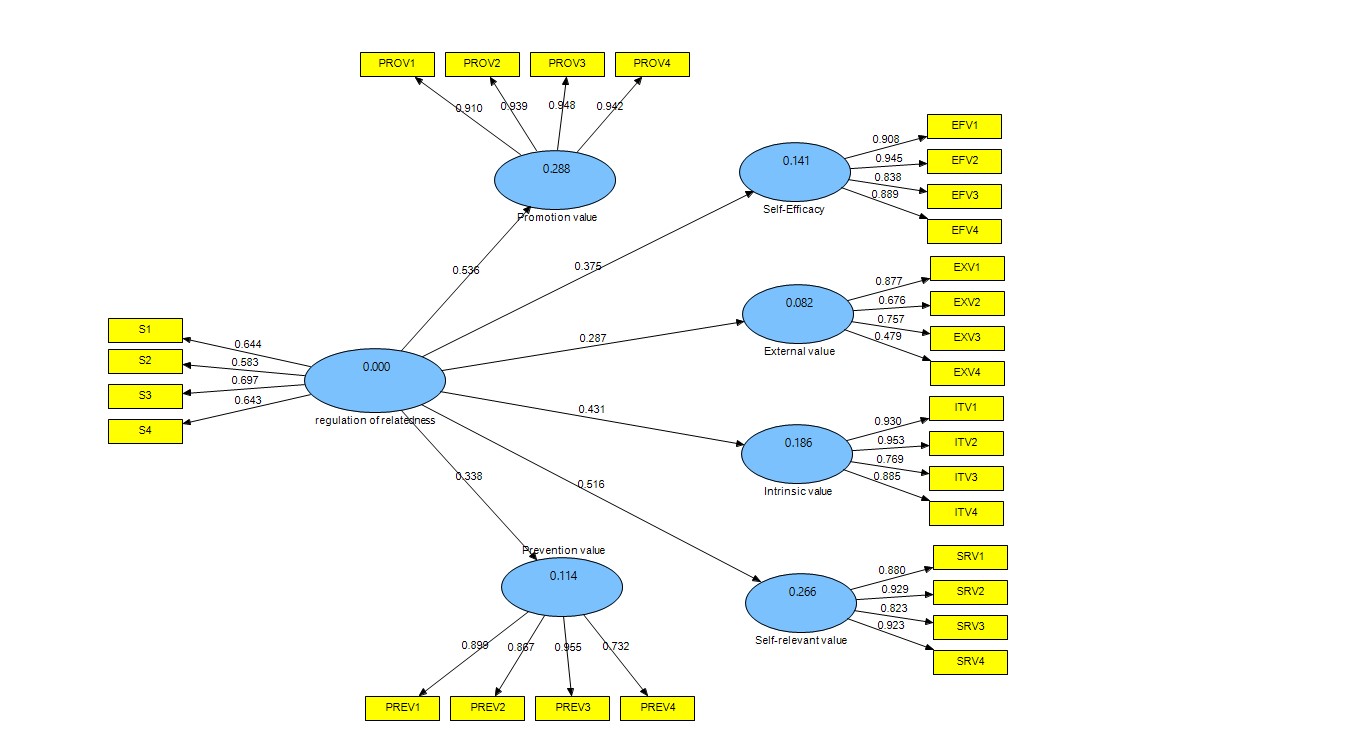


Figure 3: Structural model and predictive relationship between regulation of relatedness strategy and motivational components


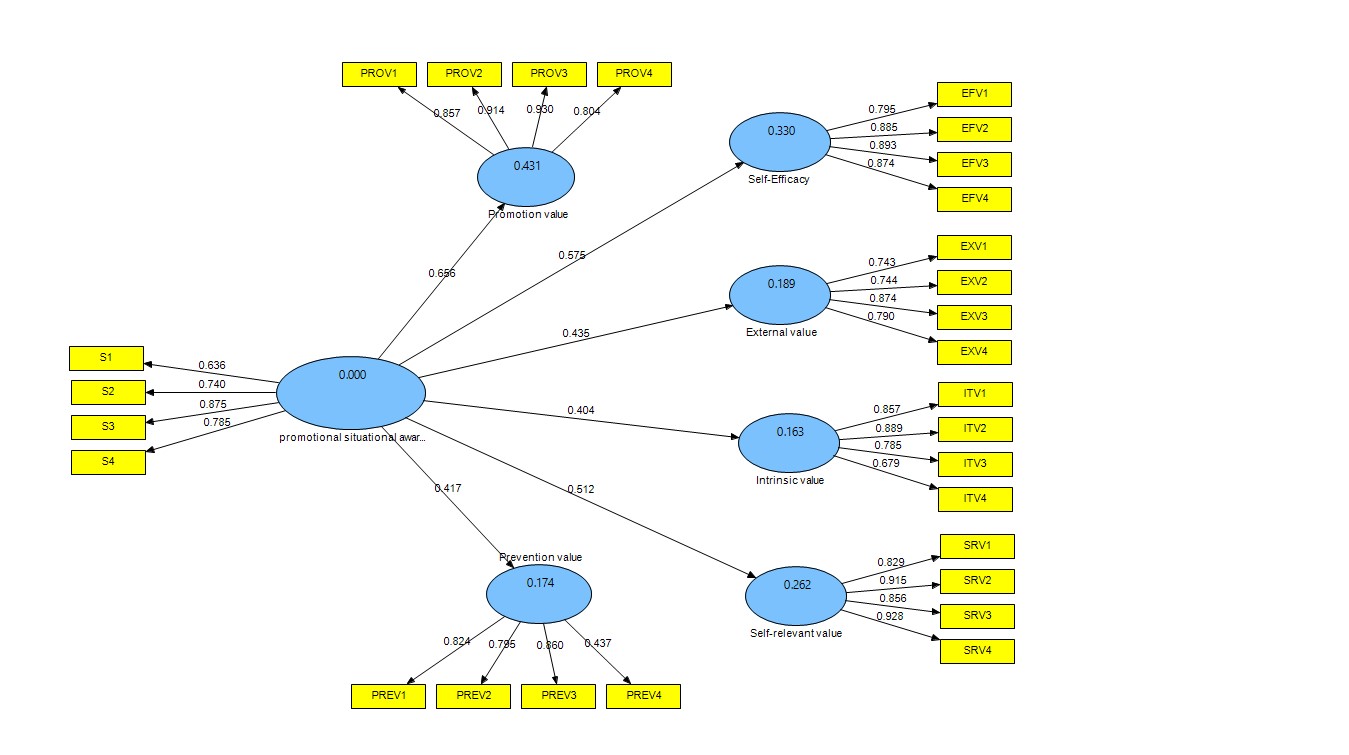


Figure 4: Structural model and predictive relationship between promotional situational awareness strategy and motivational components


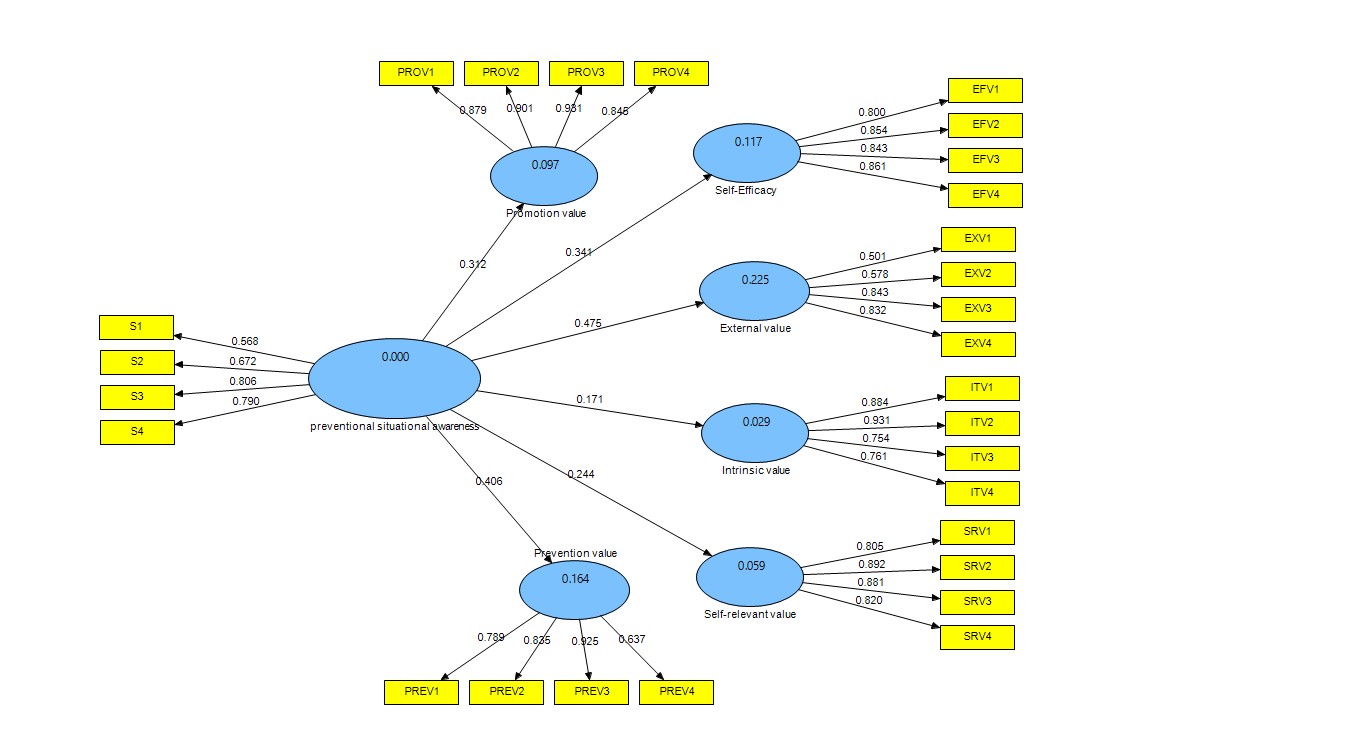


Figure 5: Structural model and predictive relationship between preventional situational awareness strategy and motivational components


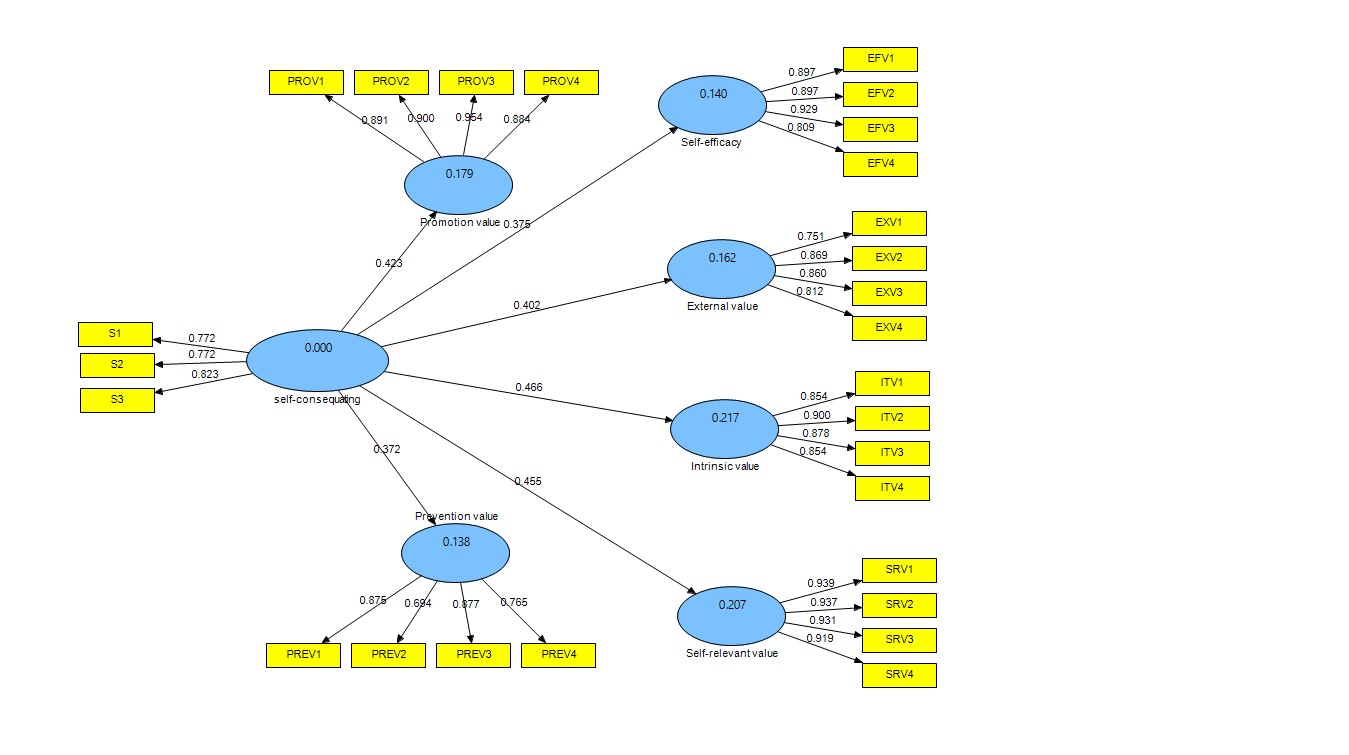


Figure 6: Structural model and predictive relationship between self-consequating strategy and motivational components


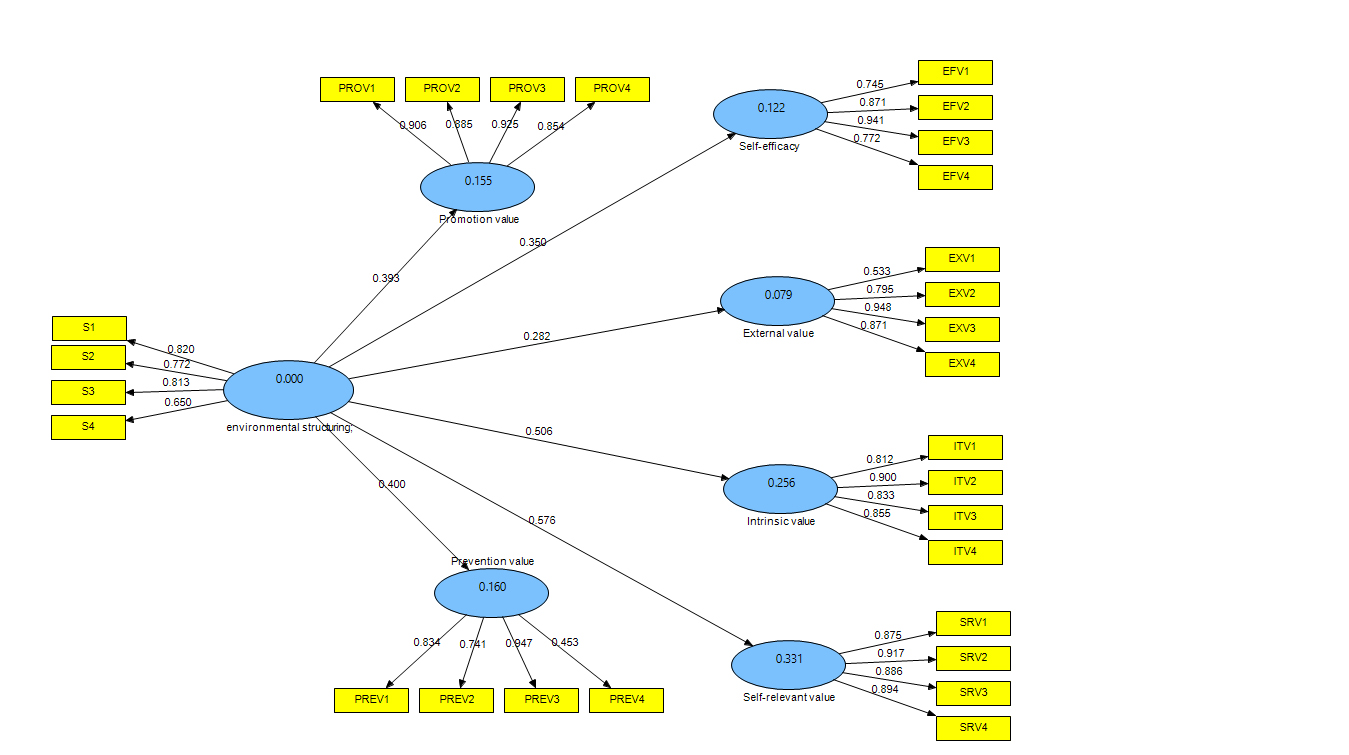


Figure 7: Structural model and predictive relationship between environmental structuring strategy and motivational components (Before deleting item PREV4)


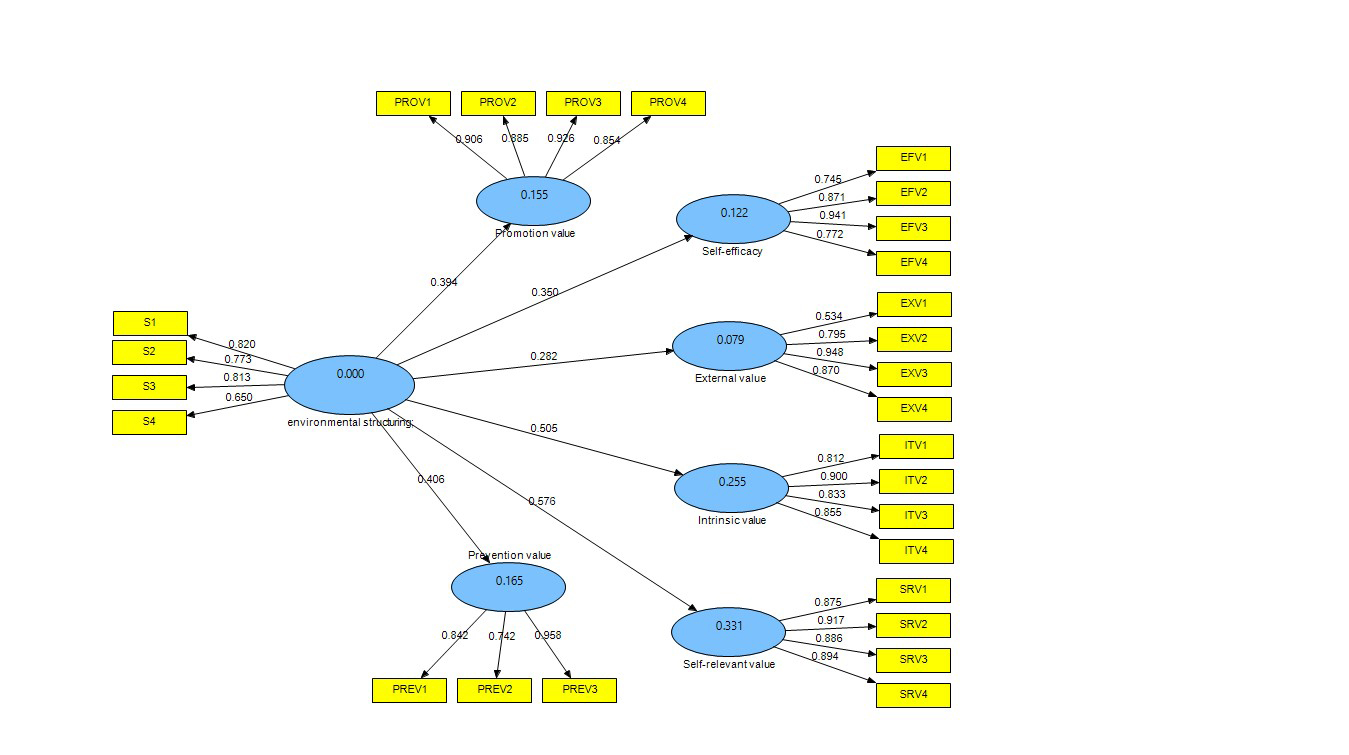


Figure 8: Structural model and predictive relationship between environmental structuring strategy and motivational components (After deleting item PREV4)
